# Toll-Like Receptor 3 (TLR3) Plays a Major Role in the Formation of Rabies Virus Negri Bodies

**DOI:** 10.1371/journal.ppat.1000315

**Published:** 2009-02-27

**Authors:** Pauline Ménager, Pascal Roux, Françoise Mégret, Jean-Pierre Bourgeois, Anne-Marie Le Sourd, Anne Danckaert, Mireille Lafage, Christophe Préhaud, Monique Lafon

**Affiliations:** 1 Neuroimmunologie Virale, Institut Pasteur, Paris, France; 2 Plate Forme d'Imagerie Dynamique, Institut Pasteur, Paris, France; 3 Département de Neuroscience, Institut Pasteur, Paris, France; University of North Carolina, United States of America

## Abstract

Human neurons express the innate immune response receptor, Toll-like receptor 3 (TLR3). TLR3 levels are increased in pathological conditions such as brain virus infection. Here, we further investigated the production, cellular localisation, and function of neuronal TLR3 during neuronotropic rabies virus (RABV) infection in human neuronal cells. Following RABV infection, TLR3 is not only present in endosomes, as observed in the absence of infection, but also in detergent-resistant perinuclear inclusion bodies. As well as TLR3, these inclusion bodies contain the viral genome and viral proteins (N and P, but not G). The size and composition of inclusion bodies and the absence of a surrounding membrane, as shown by electron microscopy, suggest they correspond to the previously described Negri Bodies (NBs). NBs are not formed in the absence of TLR3, and TLR3^−/−^ mice—in which brain tissue was less severely infected—had a better survival rate than WT mice. These observations demonstrate that TLR3 is a major molecule involved in the spatial arrangement of RABV–induced NBs and viral replication. This study shows how viruses can exploit cellular proteins and compartmentalisation for their own benefit.

## Author Summary

Viruses are obligate parasites. The progression of their life cycle depends on their hijacking the cellular metabolism and machinery. Human neurons produce TLR3, a protein involved in early host defence mechanisms and the modulation of neuronal survival. Rabies virus is a neurotropic virus, infecting mainly neurons. In this study, we showed that rabies virus exploits TLR3 function to store viral proteins and viral genomic material in particular areas of the cell where virus multiplication occurs. We found that, during the course of infection, large (1–3 µm) spherical inclusions were formed within the region around the nucleus. These inclusions were composed of an inner core of aggregated TLR3 surrounded by a coat of viral proteins and genomic material. These inclusions were revealed to be the previously described Negri Bodies (NBs). In absence of TLR3, NBs were no longer formed and virus multiplication rate decreased. Mice deficient in TLR3 were more resistant to rabies and had lower levels of infection in their brains. This study shows how neurotropic viruses, such as rabies virus, hijack normal functions of neuronal proteins and use cell compartmentalisation to promote viral multiplication.

## Introduction

Toll-like receptors (TLR) are innate immune receptors that recognise and respond to the presence of PAMPS (pathogen associated molecular patterns) encoded by pathogens [1]. TLR3 is a type I intracellular transmembrane protein that contains a large leucine-rich repeat (LRR) in the extracellular region and a Toll/Il-1 receptor homology (TIR) signalling domain in its cytoplasmic region. TLR3 can detect the presence of and respond to exogenous and endogenous RNA molecules: dsRNA of viral origin, mimicked by polyriboinosine-polyribocytidylic acid (polyI∶C); mRNA; and ssRNA (polyinosinic acid) [2],[3],[4]. Upon ligand binding, TLR3 signals via a MyD88-independent signalling pathway involving the adaptor molecule TRIF/Ticam-1 [5],[6]. TRIF can induce activation of IRF3 and NF-κB, notably through the interaction with TRAF6 and RIP1 [7],[8],[9],[10],[11],[12],[13],[14]. TLR3-dependent activation leads to the expression of genes encoding proinflammatory cytokines, chemokines and IFN-α/β.

TLR3 is present within the central nervous system (CNS) [2]. High levels of TLR3 are found in glial cells [15],[16] and neurons in disorders of the brain, neurodegenerative diseases and viral infections [17],[18]. The high levels of TLR3 in the CNS suggests an important role in the response to neuronal injury and/or viral infection [19], which may involve mechanisms other than those limited to the innate immune response. Indeed, TLR3 has been described as a negative regulator of axonal growth [20].

In non-neuronal cells (dendritic cells (DCs) or epithelial cells), TLR3 is found in intracellular compartments — sometimes small perinuclear structures (300–500 nm), such as those observed after overexpression [21],[22], or others identified as multivesicular bodies (MVBs) [16],[21]. Intracellular localisation of TLR3 appears to be crucial for its activation [23],[24]. In neuronal cells, TLR3 is mainly intracellular [16]–[18]. However, the precise intracellular localisation of TLR3 in neuronal cells is largely unknown.

Rabies virus (RABV) almost exclusively infects neurons, where it triggers interferon, inflammatory and antiviral responses [17],[25]. The virus particle binds cell-surface receptors and follows the endosomal pathway, allowing the release of viral nucleocapsid (NC) into the cytoplasm. The life cycle of the virus then progresses in the cytoplasm, with transcription of the five N, M, P G and L viral genes and the replication of negative- and positive-polarity genomes [19],[26]. Moreover, RABV infection induces the formation of cytosolic protein aggregates called Negri Bodies (NBs). NBs are characterized by the accumulation of viral NC proteins [27]–[29]; they also contain endothelial nitric oxide synthase (eNOS) [30]. NBs resemble inclusion bodies and/or aggresomes seen in several neurodegenerative disorders [31]. Aggresomes are defined as pericentriolar cytoplasmic inclusions resulting from the aggregation of misfolded and/or ubiquitinated proteins [32],[33]. The aggresomal subcellular compartment sequesters proteins produced in excess following a cellular stress such as a viral infection. These proteins are then targeted to proteasomes and/or autophagy components present in this area. Some viruses may use aggresomes to enhance replication [34],[35].

In this study, we further analysed the production, function and spatiotemporal location of neuronal TLR3, and notably its association to the endosomal compartment. We carried out these studies either in cultures of human neuronal cells (neuronal precursor Ntera-2clD/1, post mitotic neurons NT2-N or neuroblastoma cell line SK-N-SH) or in TLR3^−/−^ mice using the pathogenic RABV strain CVS.

## Results

### Human Ntera-2clD/1, SK-N-SH, and NT2-N cells express a full-length TLR3 whose expression is not altered by RABV infection

**Figure 1:**
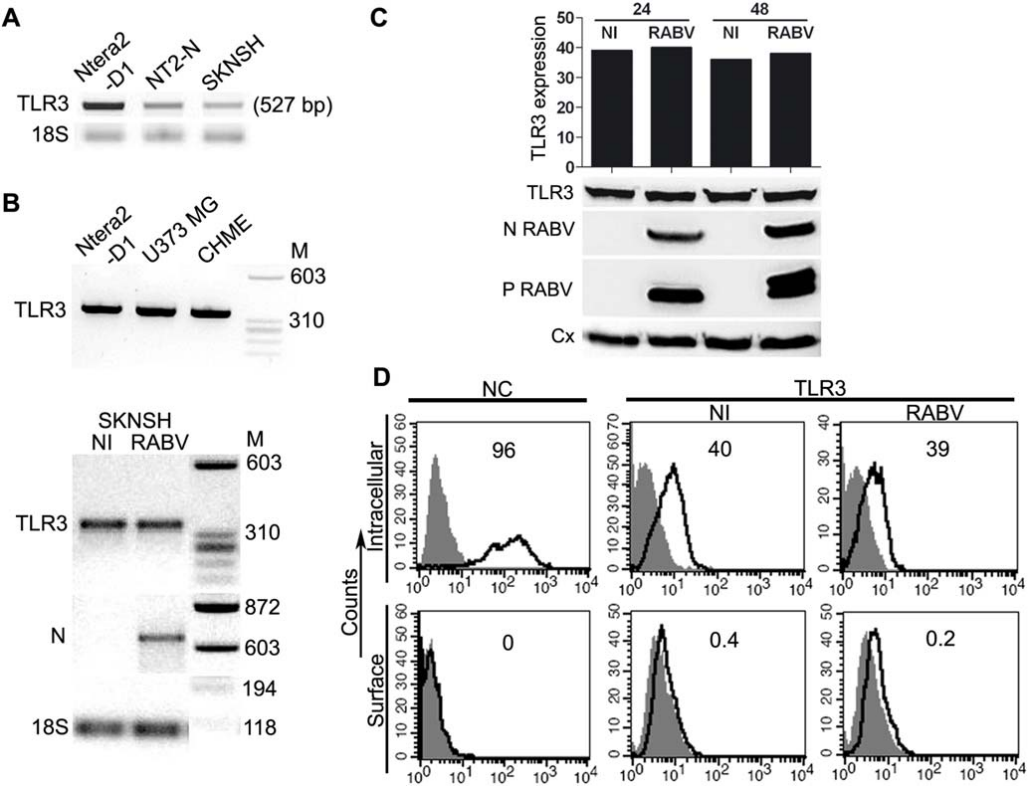
Expression of TLR3 in non-infected and RABV–infected neuronal cells. (A) Neuronal precursors (Ntera-2clD/1), human post-mitotic neurons (NT2-N), and neuroblastoma cells (SK-N-SH) express *TLR3* transcripts (size 527 bp), as shown by RT–PCR. 18S was used as a reporter gene. (B) RT–PCR was performed with a pair of primers corresponding to exon 4 of *TLR3*. The amplified fragments from Ntera-2clD/1, astrocytes (U373 MG), microglia (CHME) (upper panel), or SK-N-SH (lower panel) were a similar size (345 bp), suggesting that *TLR3* does not undergo alternative splicing in these neural cell types. Alternative splicing was not observed during infection, monitored by viral *N protein* (N) transcription (lower panel). M denotes molecular weight. (C) TLR3 protein was detected in RIPA lysates of non-infected (NI) and RABV–infected Ntera-2clD/1 cells using a mAb (cloneIMG315-A) directed to the NH2 terminal of TLR3. Infection was monitored by detection of the viral N (N) and P (P) proteins. TLR3 protein (102 kD) was not upregulated by infection, as shown by quantification of TLR3 (histogram) using calnexin (Cx) as a standard (GeneTools, Syngene). The same results were obtained for proteins extracted using Phosphosafe extraction buffer. Graph was generated using GraphPad Prism. (D) Cytofluorimetry analysis showed that TLR3 has an intracellular location and cannot be detected at the surface of SK-N-SH cells. This distribution pattern was not modified by infection. The viral nucleocapsid (NC)—left panels—is strictly intracellular, and its detection was thus used as a control for membrane impermeability. The solid line corresponds to RABV–infected cells and the histogram to NI cells. TLR3 protein (right panels) was analysed by flow cytometry using a polyclonal antibody directed to the NH2 terminal of TLR3 (Sc-Q18) in NI and RABV–infected cells. Histograms correspond to secondary Ab and solid lines to TLR3 staining. Numbers represent the percentages of cells positive for TLR3 or NC proteins. These results are representative of at least 5 separate experiments.

Human post-mitotic neurons (NT2-N) produce several TLRs, including TLR3 [17],[19]. TLR3 mRNA levels were analysed by semi-quantitative RT-PCR in the neuronal precursors Ntera-2clD/1, from which NT-2N cells are derived, and in a human neuroblastoma cell line (SK-N-SH). An amplified product of 527 bp was detected in the three types of neuronal cells (Figure 1A). We then sequenced the whole TLR3-encoding gene open reading frame by direct sequencing. We found that human neuronal *TLR3* sequence from NT2-N (sequence ID: DQ445682) was strictly identical to those previously identified in other human cells (GenBank accession: NM_003265). These findings strongly suggest that TLR3 in neuronal cells could be functional. Yang *et al*. reported the existence of a TLR3 isoform in primary human astrocytes and glioblastoma [36]. This isoform is produced by deletion of an intron-like sequence within exon IV of the gene and is co-produced with wild-type TLR3. The presence of this isoform in Ntera-2clD/1, U373MG (astrocytoma) and CHME (microglia) was tested by semi-quantitative RT-PCR experiments using pairs of primers spanning the intron-like sequence in exon IV (Figure 1B, upper gel). This isoform was not detected in the three cell lines tested. Moreover, no quasi-species of wild-type transcripts or transcripts with deleted sequences were observed after RABV infection of SK-N-SH neuroblastoma (Figure 1B, lower gel).

The effect of infection on TLR3 levels was monitored by immunoblotting using two distinct antibodies (Ab) directed against the NH2 terminal of the TLR3 ectodomain (sc-Q18 and IMG-315A). Cellular extracts were prepared from Ntera-2clD/1, either non-infected (NI) or infected with RABV for 12, 24 or 48 h. We detected a 100 kDa band corresponding to full size TLR3 protein in all cell extracts (Figure 1C data shown for IMG-315A Ab). The amount of neuronal TLR3 was not affected by RABV infection even 48 h after infection (Figure 1C). These results were consistent with those obtained by cytofluorimetry (two upper right panels of Figure 1D, data shown for Sc-Q18 Ab), with no difference in proportion of cells displaying fluorescently labelled total TLR3 observed between NI and infected Ntera-2clD/1 cells (40 and 39% respectively). In experiments performed without cell permeabilisation (surface fluorescence), TLR3 was not detected at the surface of NI or infected cells (lower right panels of Figure 1D). Thus, TLR3 is not expressed at the surface, at least at detectable levels, and infection does not cause TLR3 to relocate towards the cell surface. Similar findings were obtained with the SK-N-SH neuroblastoma (data not shown).

These data indicate that human neuronal cells produce full-length TLR3; this protein was not upregulated by RABV infection and had an intracellular localisation in both NI and infected cells.

### TLR3 has a canonical endosomal localisation in human neuronal cells

**Figure 2:**
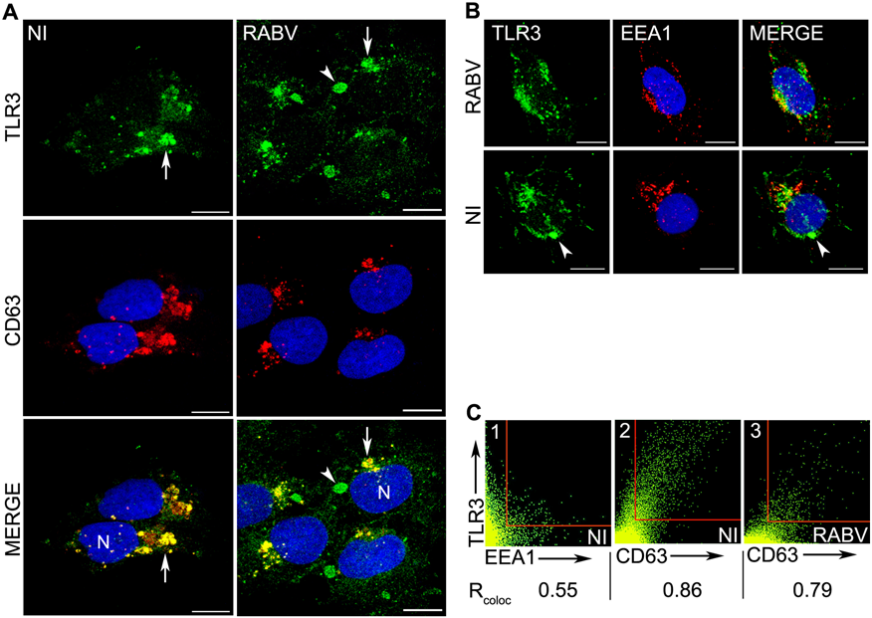
TLR3 partially colocalises with the endosomal compartment of non-infected and RABV–infected Ntera-2clD/1 cells and relocates into perinuclear aggregates after RABV infection. Co-immunostaining of TLR3 (Ab sc-Q18, green) with late (A) and early (B) endosomal compartments using anti-CD63 and anti-EEA1 Ab (red), respectively, in non-infected (NI) (A: left panels, B: upper panels) and RABV-infected (A: right panels ; B: lower panels) Ntera-2clD/1cells. Nuclei are stained with Hoechst. TLR3 colocalises with CD63 in NI (A, Merge left, arrow) and in RABV–infected cells (A, Merge right, arrow). TLR3 protein additionally forms perinuclear aggregates that do not contain endosomal markers (A and B, arrowheads). Images were acquired using a Zeiss LSM 510 META confocal microscope. Projection of 8–10 confocal images is shown. Bars = 10 µm. (C) Colocalisation of TLR3 with endosomal markers: correlation diagrams and Pearson's correlation coefficients (Rcoloc) are shown: TLR3 (Y axis) with either EEA1 (1) or CD63 (2 and 3) (X axis) are shown for NI (1–2) and RABV–infected cells (3). TLR3 shows very little association with the early endosomal compartment (1), but is strongly associated with the late endosomal marker in the absence of infection (2). Colocalisation with the late endosomal marker decreases following RABV infection (3).

Intracellular localisation of TLR3 was further investigated in Ntera-2clD/1cells by immunocytochemistry and confocal microscopy. In the absence of infection, TLR3 was found within small vesicles throughout the cytoplasm. Co-immunocytochemistry analysis using Ab directed against the NH2 terminal of TLR3 (Sc-Q18) and against either CD63, a marker of late endosomes and/or multivesicular bodies (left panels of Figure 2A), or a marker of early endosome (EEA1) (upper panels of Figure 2B) revealed that a proportion of TLR3 was associated with endosomes, mainly in late endosomal vesicles (colocalisation index of 0.55 and 0.86 for early and late endosomal markers, respectively; Figure 2C). A small proportion of TLR3 was found associated with Golgi (GM130) and with the endoplasmic reticulum, ER (calnexin) (data not shown).

Thus, in neuronal cells, TLR3 protein was located mainly within the early/late endosomal compartment.

### As well as its endosomal localisation, TLR3 targets large perinuclear aggregates in RABV–infected cells

Two days after RABV infection, the endosomal distribution of TLR3 was conserved (as shown in Figure 2A, left panel, and Figure 2B, lower panel). However, colocalisation coefficients between TLR3 and CD63 were slightly lower in RABV-infected than in NI cells (0.86 and 0.79 for NI and RABV-infected cells, respectively). This decrease may result from the appearance of a new site of TLR3 intracellular localisation. TLR3 became concentrated in large perinuclear aggregates in RABV-infected cells (arrow heads in Figure 2A and 2B). This observation was reproduced using three distinct TLR3-specific Ab (Sc-Q18, Sc-C20 and FITC-conjugated Ab IMG-315C) and in cultures of NT2-N (Figure S1) and SK-N-SH (data not shown). Perinuclear aggregates were not stained by anti-CD63 or anti-EEA1 Ab, or by OKT9, a marker of recycling endosomes. This suggests that the RABV-induced aggregates were distinct from the endosomal compartment. RABV-induced TLR3 aggregates were not associated with Golgi (GM130) or the ER (calnexin) (data not shown). This aggregation of TLR3 was not observed when cells were infected with herpes simplex virus type 1 or Sendai virus, or when cells were treated for 24 h with rIFN–β (500 IU/ml) (data not shown).

These observations suggest that RABV triggers perinuclear aggregation of TLR3, distinct from the endosomal compartment.

### TLR3 proteins are associated with RABV N and P proteins in perinuclear inclusions formed by an inner TLR3 core coated with viral proteins

**Figure 3:**
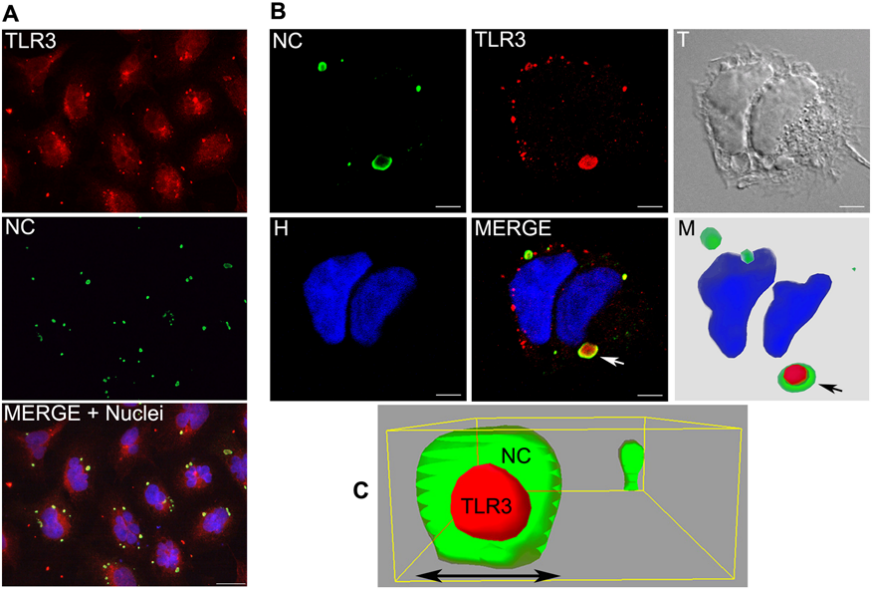
TLR3 and viral NC proteins are assembled in spherical perinuclear structures. (A) RABV-infected Ntera-2clD/1 cells were co-stained with Ab directed against the viral nucleocapsid (NC green), TLR3 (Ab Sc-C20, red), and Hoechst (nuclei, blue). In all infected cells TLR3–positive aggregates colocalize with viral NC forming perinuclear structures (Merge, lower panel). Bar = 10 µm. (B) RABV-infected Ntera-2clD/1 cells were co-stained with Ab directed against the viral nucleocapsid (NC green), TLR3 (Ab Sc-C20, red), and Hoechst (nuclei in blue). T is the transmission picture. The merged picture shows that NC accumulates with some perinuclear TLR3–positive aggregates (arrow). These perinuclear aggregates are visible in the transmission image. M is the 3D rendering (Imaris, Bitplane AG) after deconvolution (Huygens, Scientific Volume, Imaging). TLR3 constitutes the core of the structure (red internal core), surrounded by a coating of viral NC (green halo). Nuclear material was not present in these structures. Bar = 5 µm. (C) Enlargement of a 3D rendering of a TLR3–containing aggregate, showing the typical organisation of RABV–induced inclusions bodies composed of an inner core (TLR3 in red) surrounded by a viral NC protein cage (green halo). Diameter of the aggregate is 2.7 µm.

**Figure 4:**
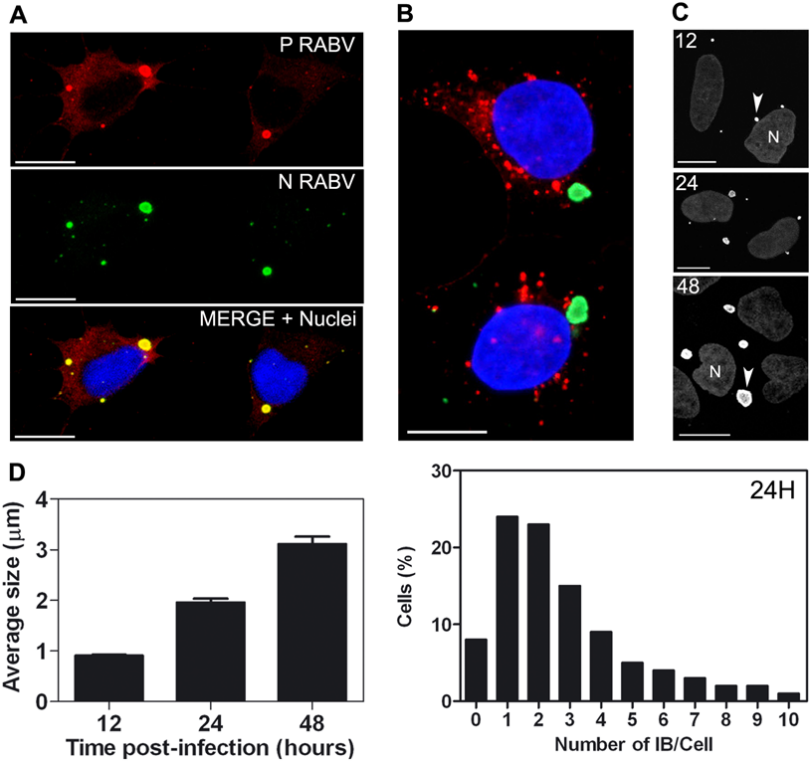
RABV–induced inclusion bodies correspond to Negri Bodies (NBs). They contain viral N and P, but not G. (A) Immunostaining of RABV P protein (P) and N protein (N) using specific Ab shows the colocalisation of the two proteins within the NBs (merge+nuclei) (B) RABV–induced aggregates (NC, green) do not contain RABV G protein (G, red). Bar = 10 µm. (C–D) Ntera-2clD/1 cells were infected with RABV, fixed with PFA, and stained with an Ab directed against NC 12, 24, and 48 h pi. (C) Small perinuclear aggregates are detected as early as 12 h pi and their size increases with time. (D, left panel) Size of aggregates increase as the infection progress from a 1 µm diameter 12 h pi up to 3.0 µm 48 h pi. In this experiment, viral NB diameters were measured manually using the Leica FW 4000 software. Graph represents means with SEM. (D, right panel) 24 h after pi majority of cells contains 1–3 NBs. Viral NB were quantified by Acapella Software.

Confocal microscopy analysis of RABV-infected cells co-stained with Ab directed against TLR3 and RABV nucleocapsid NC revealed that RABV NC was located in perinuclear inclusions, with the great majority displaying the same localisation pattern as TLR3-containing aggregates (Figure 3A, TLR3 in red, NC in green,). Viral NC (comprising N, P, L proteins and the viral RNA genome) seemed to form a ring around a TLR3 core. TLR3-positive inclusions were further analysed by confocal microscopy (Figure 3B) and imaging (Figure 3C). Modelling of deconvoluted confocal images with 3D Imaris® software confirmed the particular spatial organisation of TLR3/NC aggregates (Figure 3C). The reconstruction of images of a typical 48 h TLR3/NC aggregate is shown in Figure 3B. The round-shaped perinuclear structure has a total diameter of 3.0 µm with an NC coating 0.5 µm thick. TLR3/NC aggregates were also observed using monoclonal antibody (mAb) specific for viral P or N protein, confirming that the main protein constituents of NC were present in the aggregates (Figure 4A). In contrast, immunocytochemistry performed with a mAb directed against the RABV G protein — associated with the RE or Golgi vesicles in the cells — showed that TLR3 aggregates were not associated with RABV G protein (Figure 4B). Co-immunoprecipitation performed with Ab directed against NC and TLR3 (Q18 and C20 Ab) gave negative results (data not shown), suggesting that interactions between TLR3 and viral NC were either weak or indirect.

The kinetics of RABV NC aggregate formation were analysed in Ntera-2clD/1 at 12, 24 and 48 h post-infection (pi) and stained with anti-NC Ab (Figure 4C). NC aggregates were detected as early as 12 h pi, with an average diameter of 1.0 µm (Figure 4E). The size of aggregates increased as infection progressed, reaching an average size of 3.0 µm by 48 h pi (Figure 4D, left panel). Number of inclusions per cell was analyzed by Acapella software in 24 h RABV-infected SK-N-SH cells (Figure 4D, right panel). Majority of cells harbour one or two perinuclear TLR3 aggregates 24 h after infection. Later in the infection (48 h after infection and thereafter), the average number of inclusions per cell increases (as described below), suggesting that NB formation is a highly dynamic process.

These observations suggest that RABV triggers formation of perinuclear TLR3 aggregates -surrounded by a halo of viral N and P proteins- which number and size are modulated during infection.

### TLR3/NC aggregates contain the RABV viral genome and are Negri Bodies (NBs)

**Figure 5:**
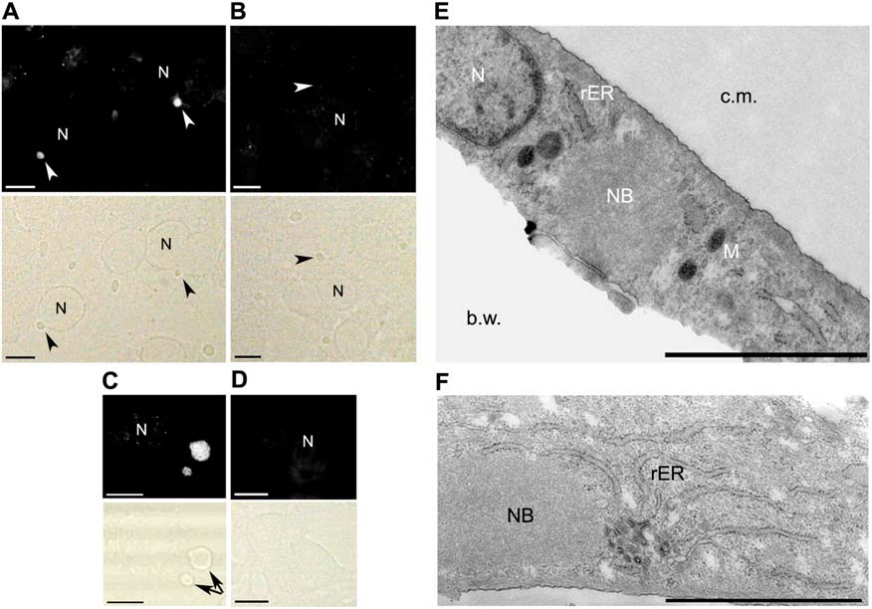
NBs contain viral genomic material, suggesting they are specialized sites for viral multiplication. Aggregates contain RABV genomic material. RABV–infected (A–C) and non-infected (D) BSR cells were incubated with (A, C, D) or without (B) fluorescein-conjugated RNA probes specific for the viral RABV genome. Both fluorescent (upper panels) and transmission images (lower panels) were shown for each condition. (A) Genomic material was detected in the discrete aggregates (arrows) located around the nuclei (N) of RABV–infected cells. (B) No fluorescence is seen in RABV–infected cells in the absence of RNA probes. These pictures were acquired with a Leica DM5000B fluorescence microscope. (C) Detail of a RABV–infected single cell with the anti-genomic probe (confocal stack). (D) No fluorescence was detected in non-infected cells incubated with the fluorescein-conjugated RNA probes. Bars (A–D) = 10 µm. (E and F) Neuroblastoma SK-N-SH cells were infected for 48 h with RABV, fixed and prepared for electron microscopy analysis. (E) Electron microscopy of a section showing that aggregates are located near the nucleus (N) surrounded by mitochondria (M) and rough endoplasmic reticulum (rER). Aggregates are not limited by a membrane. B.w = base of culture well. C.m = culture medium. (F) Viral inclusions are seen close to the sites of viral assembly. Nests of virus particles (arrow) are located close to viral NBs and rER. Bars = 2 µm.

TLR3 has been described for its ability to bind viral RNA. We carried out hybridisation experiments using a fluorescent probe specific for the RABV genome leader sequence to determine whether the TLR3/NC aggregates contained viral RNA (Figure 5). We did not detect fluorescence in the absence of probe (Figure 5B) or in NI cells (Figure 5D). The fluorescent probe stained perinuclear structures (Figure 5A–5C), corresponding to TLR3/NC aggregates, forming discrete ovoid structures visible in transmission images, as described above (Figure 3). Intensity of fluorescence was variable, suggesting that accessibility of the viral RNA may differ among aggregates. These observations show that TLR3/NC aggregates also contain RABV genomic material. Immunocytochemistry experiment using J2 mAb specifically recognizing dsRNA [37],[38],[39]—including endogenous cellular dsRNA [40],[41]—revealed accumulation of dsRNAs in NBs (Figure S2).

The presence of viral N and P proteins and genomic material within the aggregates suggests that they could be NBs. NBs are found both in the neurons of rabid brain and *in vitro* in RABV-infected neuronal cells, in the form of accumulated viral material around the nucleus. Electron microscopy analysis of ultra-thin sections of SK-N-SH 48 h after RABV infection showed that amorphous aggregates (usually one or two per cell), with an average diameter of 3.0 µm and without a membrane, were indeed present and displayed perinuclear localisation (Figure 5E). We did not find such aggregates in NI cells (data not shown). We used electron microscopy to analyse cells treated with Triton X-100 (0.1%) and stained with Ab directed against NC or TLR3; cellular structures were not preserved. Aggregates were strongly stained (DAB accumulation) with both Ab, indicating they contained both TLR3 and RABV NC proteins (Figure S3).

Electron microscopy also showed that the aggregates were located in particular cell areas characterised by the accumulation of surrounding mitochondria (M), rough endoplasmic reticulum (rER) and, in some cells, ‘nests’ of viral particles (Figure 5F). These areas, with their particular composition resembled the viral factories previously described for other viruses. Thus, TLR3/NC aggregates may function as a storage or construction area for viral NC.

Overall, these observations suggest that TLR3/NC aggregates observed in RABV-infected neuronal cells correspond to previously described NBs.

### Are NBs virus-induced aggresomes?

**Figure 6:**
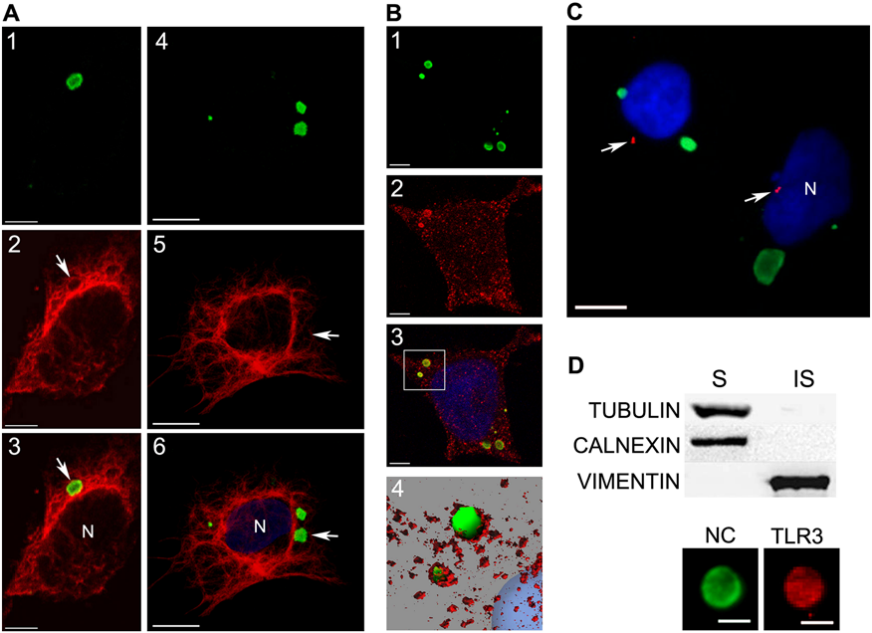
RABV–induced NBs exhibit some characteristics of aggresomes. (A) RABV–infected cells were immunostained with Ab against viral NC (1 and 4) and co-stained with either anti-pan-tubulin (2 and 3) or anti-vimentin Ab (5 and 6). Merged images are shown in 3 and 6. NBs (arrows) are associated with tubulin fibres (3); however, in contrast to canonical aggresomes, NBs are not surrounded by a ‘cage’ of the intermediate filament protein vimentin (6). N = nucleus. Bars = 10 µm. (B) RABV–infected cells were co-stained with Abs directed against viral NC (1) and anti Hsp70 (2). NBs are associated with the chaperone Hsp70, as shown by confocal analysis and 3D modelling [3 is the merged image; 4 is a 3D rendering (Imaris®, Bitplane AG) after deconvolution (Huygens, Scientific Volume, Imaging) of the perinuclear area (white square) from 3]. Bar = 5 µm. (C) NBs (green) are not formed at the MTOC/centrosome, detected using an antibody directed against γ-tubulin (arrows). N = nucleus. Bar = 10 µm. (D) RABV–infected cell lysates were separated after detergent treatment, into soluble (S) and insoluble (IS) fractions. The IS fraction contained the insoluble cytoskeletal protein vimentin but not tubulin or calnexin (Western Blot, upper panel). The IS fraction also contained NBs, which showed positively with anti-NC (green) and anti-TLR3 Ab (red). Bars = 2 µm.

Due to their perinuclear localisation, size and protein composition, it is possible that NBs represented virus-mediated aggresomes. Aggresomes are associated with molecular chaperones such as Hsp70 and surrounded by a cage of cytoskeleton proteins such as vimentin and α-tubulin [32],[33],[42]. Thus, we studied the presence of aggresome characteristics in NBs. Examination of the distribution of α-tubulin and vimentin in RABV-infected cells revealed that a ring of α-tubulin indeed surrounded the NC-positive aggregates (Figure 6A, 1–3). However, vimentin was not closely associated with NBs (Figure 6A, 4–6). The chaperone Hsp70 was present at the periphery of the NBs (Figure 6B). Aggresomes are often formed at the microtubule organising centre (MTOC), which can be visualised using Ab directed against γ-tubulin [43]. Double staining of MTOC and NC proteins clearly demonstrated that NBs are not localized at the MTOC (Figure 6C).

When cells are lysed in the presence of detergent, aggresomes are retained in the insoluble (IS) fraction, together with cytoskeletal proteins such as vimentin, whereas proteins in the cytoplasm or associated with the ER — such as calnexin — appear in the soluble (S) fraction. S and IS fractions were prepared from Ntera-2clD/1 cells infected with RABV for 48 h, and were separated by SDS-PAGE. The IS fraction prepared from RABV-infected cells did not contain calnexin or α-tubulin. These proteins were present in the S fraction, as expected, with the IS fraction containing vimentin (Figure 6D, upper). Immunostaining revealed discrete NBs (positively stained for both RABV NC and TLR3) in the IS fraction (Figure 6D, lower), indicating that NBs are detergent-resistant structures.

**Figure 7:**
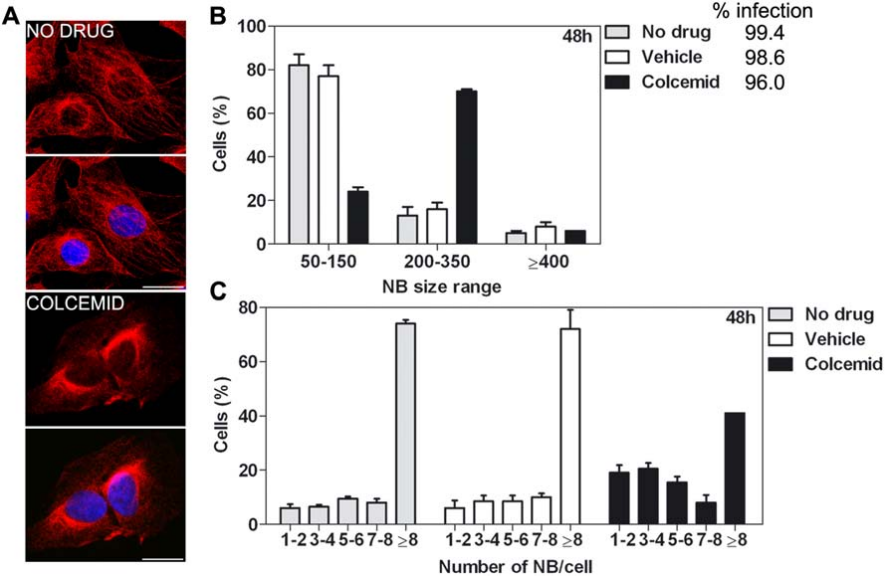
Involvement of the microtubule network in NBs dynamics. To analyze the role of microtubules in the NBs outcome, 24 h RABV–infected SK-N-SH cells were either treated with colcemid (a drug which disrupts microtubule network), with PBS (vehicle), or in the absence of drug for 24 h. (A) Efficiency of colcemid on microtubules stability was assayed by staining 24 h PBS–treated (upper panels), and colcemid treated cells (lower panels) with an Ab directed against tubulin (red). Nuclei are stained with DAPI (blue). Bar = 10 µm. (B and C) Effect of colcemid on size and number of NBs per cell in 48 h RABV–infected cells. After fixation, cells were stained with viral NC Ab. Size (units = pixels) and number of viral inclusions were determined and analysed using Acapella software. Infection levels were similar in presence or in absence of colcemid. (B) Colcemid treatment modifies the size of NBs in the culture by increasing the number of NBs of intermediary range size (200–350 pixels). (C) Colcemid treatment interrupts the increase of NBs per cell (>8 NBs/cell) seen 48 h after infection. In absence of treatment 75% of 48 h RABV infected cells contains >8 NBs per cell. This percentage is reduced by one half (35–40%) after a 24 h colcemid treatment. Graphs represent means and SD (standard deviation).

Given the major role of microtubules in the formation of aggresomes, we analysed their role in the formation of NBs. We treated RABV-infected SK-N-SH cells (24 h pi) with colcemid (Figure 7A) — an inhibitor of microtubule polymerisation — diluted in PBS vehicle, or with vehicle alone or without any reagent. Effect of colcemid on size and number of NBs per cell was analyzed 24 h post treatment -corresponding to 48 h post infection- (Figure 7B and 7C). As shown in Figure 7B, colcemid treatment modified the size of NBs. Overall, in colcemid-treated cells there are more cells with large NB (range between 200 and 350 pixels) than cells exhibiting small NB (range 50 to 150 pixels). In the meantime, colcemid treatment drastically modified the distribution of NB per cell (Figure 7C). In absence of treatment, 24 h after infection RABV-infected cells harboured 2 or 3 NBs per cell (as shown in Figure 4D). This number rises up to more than 7–8 NBs per cell 48 h after infection (Figure 7C). Colcemid treatment blocks this increase since colcemid treated cells have less NBs per cell than non-treated cells. These observations indicate that microtubule polymerisation play a role in the dynamic of NBs particularly after their formation, as suggested by the increase in number of NBs per cell between 24 and 48 h pi. These data support a role of microtubules in the outcome of NBs. NBs from cells treated with colcemid were twice as small as those from cells treated with vehicle alone (Figure 7B), supporting a role for microtubules in NB formation.

Overall, our findings showed that NBs are resistant to detergent, involve microtubules in their outcome and are surrounded by the cellular α-tubulin and chaperone network. These features are reminiscent of aggresomes. However, NBs were not associated to the MTOC and not surrounded by a vimentin ‘cage’. Thus, they do not represent canonical aggresome structures.

### TLR3 is necessary for the formation of NBs

**Figure 8:**
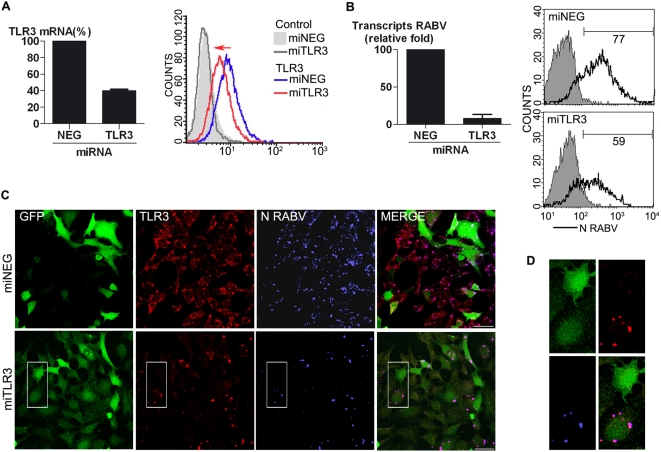
TLR3 is required for the formation of viral NBs. Hek293A cells were nucleofected (Q-001 Amaxa Program) with Emerald-GFP-plasmids encoding specific miRNA for TLR3 (miTLR3) or control miRNA (miNEG). 24 h post-nucleofection cells were infected or not with RABV. (A) Efficiency of silencing was assayed at transcriptional level by quantitative PCR (left panel) and at protein level by cytofluorimetry (right panel). TLR3 mRNA levels in miTLR3–treated cells were 60% lower than in miNEG cells. Cytofluorimetry analysis showed that TLR3 levels (shift to the left) in miTLR3–treated cells were lower than in miNEG cells. The values are representative of at least 3 experiments. (B) TLR3 silencing decreases viral multiplication. Control cells (miNEG) and TLR3–silenced cells (miTLR3) were assessed for viral genomic material (left panel, Q-PCR) and RABV protein N levels by flow cytometry (right panel). RABV transcription was decreased by 80% after silencing panel and the percentage of cells producing N was reduced following TLR3 silencing. (C) The absence of NBs was demonstrated by immunofluorescence. TLR3–silenced cells were immunostained with anti–TLR3 (Sc-Q18) (red) and anti-viral N protein Ab (blue). Plasmid encodes emerald-GFP (green). NBs were detected in TLR3-positive cells but not in TLR3–silenced cells as observed in the miTLR3 population. Bars = 20 µm. (D) Enlarged area of C panels.

TLR3 was found at the centre of NBs. Thus, we analysed the involvement of TLR3 in the formation of NBs in *TLR3* silencing experiments. Plasmids encoding emerald GFP and miRNA targeting the TLR3 gene (miTLR3) were delivered by nucleofection (Amaxa program Q-001) into Hek293A Qbiogene cells. Non-specific miRNA (miNEG) was used as a control. Nucleofection efficiency (≥90%) was controlled using a plasmid encoding the GFP protein (provided by the Cell Nucleofector Kit V, Amaxa). Cells were infected with RABV 24 h after nucleofection. 48 h pi, RNA was extracted or cells analysed by immunocytochemistry. TLR3 mRNA levels were compared between miTLR3 and miNEG cultures. As shown in the left panel of Figure 8A, the level of TLR3 mRNAs was decreased by 60% in miTLR3 compared to miNEG cells. Cytofluorimetry analysis of TLR3 (sc-Q18 Ab) stained cells showed that these reduced mRNA levels were associated with a reduced intensity of fluorescence (Figure 8A, right panel), indicative of reduced TLR3 protein production in miTLR3 cultures. Moreover, the reduced TLR3 levels were accompanied by a 10-fold reduction of viral transcription (Figure 8B, left panel) and RABV N protein expression as shown by cytofluorimetry (Figure 8A, right panel), with N protein present in 77% of miNEG cells and 59% of miTLR3 RABV-infected cells.

Transitory nucleofected cells with the miTLR3 and miNEG vectors expressing emerald-GFP were assayed for expression of Em-GFP fluorescence by FACS (data not shown) and immunocytochemistry (Figure 8C), showing that the intensity of Em-GFP fluorescence is not homogenous among cells in the same population. Similar data were obtained on stable cell lines selected for resistance to blasticidin which all possess the expression plasmid but nevertheless exhibit a heterogenous Em-GFP fluorescence (data not shown). Cells were immunostained with anti-TLR3 (red) and anti-N-RABV mAb (blue) showing that the miTLR3 population of cells exhibits some TLR3-negative cells (Figure 8C, lower panel) in contrast to the miNEG population (Figure 8C, upper panel). In the TLR3-negative cells, NB formation is abolished. Immunocytochemistry analysis of individual cells (such as the green GFP cell on the right in Figure 8D) revealed that NBs — stained red for TLR3 and blue for RABV N protein — were not present in miTLR3 cells. These observations strongly suggest that TLR3 is required for NB formation and RABV N expression. To determine whether the cytoplasmic domain of the TLR3 protein encompassing the signalling TIR domain was involved in NB formation, we stable transfected HEK cells with a plasmid encoding a TLR3 gene with a deleted cytoplasmic domain (pZero-hTLR3-HA). Cells transfected with the pZERO-hTLR3 plasmid are therefore unable to signal via TLR3 because the absence of the TIR domain does not allow binding of TRIF to TLR3. As shown in Figure S4B, NBs were still formed and viral transcription was not modified in pZERO-hTLR3-expressing cells (transitory experiments). Similar data were obtained with stably transfected cell lines selected for their resistance to puromycin (data shown in Figure S4 are representative of both conditions). Furthermore, the adaptor TRIF could not be detected in NBs (Figure 4C), suggesting that activated TLR3 molecules were not present in NBs.

Thus, the ectodomain of TLR3 appears to be crucial for the formation of NBs. TLR3 may therefore favour virus multiplication.

### TLR3^−/−^ mice have reduced susceptibility to rabies

**Figure 9:**
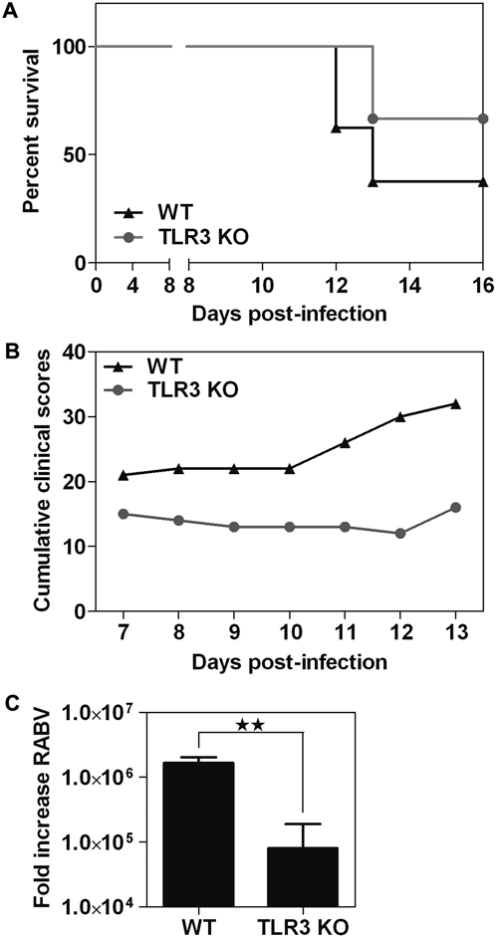
Rabies is less severe in TLR3^−/−^ mice and neuroinvasiveness is reduced. Comparison of RABV infection in TLR3^−/−^ and C57Bl6 (WT) mice. (A) Kaplan-Meier survival curves for TLR3^−/−^ (gray curve and circles) and WT (black curve and triangles) mice after injection of RABV (n = 8 WT and n = 9 KO mice). Chi-square = 2.24 (<3,6) with a degree of freedom of 1 and a significance of P (0.134). The hazard ratio was 0.2882. (B) Cumulative clinical scores are significantly different at P value<0.05. (C) Viral load (RABV genome) was measured by relative Q-PCR at day 11 pi in the brain of TLR3^−/−^ and WT mice (three mice each). Histogram represents means with SD. P value = 0.002. Two separate experiments were performed.

Assuming that TLR3-NBs are required for virus multiplication, we would expect RABV infection to be impaired in the brain of TLR3-deficient mice (TLR3^−/−^) and subsequently these mice would be expected to have a higher survival rate than WT mice. We compared the progression and outcome of RABV infection in TLR3^−/−^ with WT mice. TLR3^−/−^ (n = 9) and control WT (n = 8) C57Bl6 mice were injected with a dose of virus expected to kill 3/4 of the mice. Mice were observed for 15 days post infection. At day 13 pi, only 37% of the WT mice were alive, whereas 66% of the TLR3^−/−^ mice survived (Figure 9A). Progression of the disease in the two groups of mice is illustrated by the cumulative clinical curves (Figure 9B). Clinical score was significantly decreased in TLR3^−/−^ mice compared to WT mice. Finally RABV neuroinvasiveness was compared in these mice by measuring the amount of RABV genome in the brain of RABV-infected WT and TLR3^−/−^ mice at day 11. Levels in NI brain were given a reference value of 1, for calibration. RABV genome level in TLR3^−/−^ brain was markedly lower than in WT mice (20 times lower) (Figure 9C). All together, these strongly support a role of TLR3 in RABV infection.

## Discussion

Our study demonstrates that TLR3 is localised in the cytoplasm of non-infected neuronal cells, where it is associated mainly with the endosomal compartment. TLR3 subcellular localisation is notably altered by RABV infection, with TLR3 found within viral-induced inclusions identified as RABV-induced Negri Bodies (NBs). The presence of TLR3 in the core of viral NBs, surrounded by a ring of viral N and P proteins, appears to be crucial for NB formation. This finding demonstrates a novel function of TLR3, a molecule mostly known for its immune functions.

In the absence of infection, reactivity with anti-CD63 antibody and weak signal from EEIA-expressing early endosomal structures showed neuronal TLR3 to be confined to the endosomal compartment, associated mainly to the late endosomes and multivesicular bodies. TLR3 is rarely detectable within the Golgi apparatus or at the endoplasmic reticulum. Indeed, we observed the canonical distribution pattern of TLR3 in human non-infected neuronal cells, as previously described for human DCs or fibroblasts [21],[22].

The subcellular distribution of TLR3 is markedly altered in RABV-infected cells, being localised in perinuclear inclusion bodies, the NBs. These NBs contain viral NC but do not include the envelope viral proteins [27]. Relocation of TLR3 during viral infection has only previously been described for respiratory syncitial virus (RSV). In epithelial cells, RSV infection increases the TLR3 levels and targets TLR3 to the membrane [44]. In neuronal cells, RABV infection did not increase TLR3 protein levels, or target TLR3 to the membrane. Given that RABV infection did not increase the total pool of TLR3 protein, it is possible that the TLR3 is recruited from pre-existing cytoplasmic-, ER- or endosome-associated TLR3 by NBs. However, NBs were not stained by endosomal or ER markers, suggesting that endosome- or ER-associated TLR3 is not involved in NB formation.

NBs observed by electron microscopy did not reveal the presence of any membrane surrounding the NBs. Thus, it is unlikely NBs are autophagolysomes or lysosomes. Confocal microscopy and 3-D imaging revealed that NBs have a highly organised structure, with a TLR3-containing core surrounded by a halo of viral N and P proteins. The central position of TLR3 suggests that NB formation may be initiated by the aggregation of TLR3 molecules. A primordial role of TLR3 in NB formation is consistent with the absence of NBs from cells in which *TLR3* had been silenced. TLR3 is a horseshoe-shaped solenoid with 23 leucine-rich repeats (LRRs) located in the ectodomain of the molecule. Similarly to other solenoid proteins including polyQ, TLR3 may have an intrinsic capacity to form aggregates [45]. NBs formed even in the absence of the cytoplasmic domain of TLR3, suggesting a major role for the TLR3 ectodomain in NB formation (Figure S4). PolyQ proteins such as mutant huntingtin protein form inclusions consisting of an inner dense core of aggregated huntingtin surrounded by a ring structure composed of sequestered cellular proteins [46],[47]. Experiments involving the sequential expression of polyQ followed by expression of these cellular proteins led to the conclusion that the ring structure results from the subsequent recruitment of cellular proteins at the exterior surface of an initial polyQ core aggregate. The organisation of NBs may follow a similar pattern. In this case, NBs would result from the initial association of TLR3 molecules followed, in a second step, by the accumulation of viral proteins, forming a ring into which cellular proteins could also be inserted. This would be consistent with the concentric organisation of NB structure and observation of the cellular protein eNos is recruited in NBs in RABV-infected brain. However, the NB structure is distinct from polyQ-initiated aggregates. In particular, TLR3 in NBs is still accessible to Ab. This has not been observed for huntingtin. These observations suggest that the viral ring of protein in NBs is more porous than the ring surrounding the ordered huntingtin aggregate structure. Consequently, NB and polyQ aggregates may have distinct properties. The recruitment and sequestration of cellular proteins in polyQ aggregates may lead to the functional depletion of cellular functional proteins, possibly underlying toxic properties of these aggregates in the cell. This is not necessarily the case for NBs.

The nature of the interaction between TLR3 and viral proteins in NBs remains unclear, since attempts to coimmunoprecipitate TLR3 and NC have failed.

NBs mimic aggresomes but do not exhibit the full set of characteristics of these stress-induced cellular processes. NBs are associated to the chaperone Hsp70 and to the microtubule network, reminiscent of the two principle features of aggresomes [48]. However, the marked redistribution of vimentin fibres observed for example in GFP-250-induced aggresomes, and in Theiler's or reovirus-induced aggresome-like inclusions [49],[50] was not observed in RABV-infected neuronal cells. In contrast to most aggresomes, such as those formed in HSV2 infection [51], NBs were not associated with the microtubule organising centre (MTOC).

The effect of inhibition of microtubule depolymerisation, by addition of colcemid, on the distribution and size range of NB may indicate that microtubules are required for NB outcome. The finding that NBs do not display all the characteristics of aggresomes has also been observed for inclusions formed in African swine fever virus (ASFV), which have been described to function as “viral factories”. Viral factories are areas of cytoplasm where viral components and cellular components supporting viral replication are concentrated [51],[52]. The possibility that NBs function as dynamic structures involved in viral multiplication, as proposed by Lahaye *et al*. (submitted), is supported by electron microscopy pictures showing that NBs are found closely associated with newly synthesised viral particles. TLR3 would thus be an essential component for virus multiplication. Consistent with this notion, we showed that NBs were absent from cells with *TLR3* silencing and that TLR3^−/−^ mice were less severely infected by RABV than WT mice. Further studies are now required to show whether RABV production is promoted by TLR3.

A major function of TLR3 is to sense and respond to viral infection. The presence of TLR3 in the core of NBs can be seen as an attempt to inactivate TLR3 function. Sequestration of TLR3 into NBs could reduce the cellular innate immune response. The absence of TRIF –adaptor of TLR3- in NB could support the hypothesis that TLR3 molecules in NB are inactive-. The presence of viral RNA in NBs may reflect an interaction of TLR3 with viral components at an early stage of infection, when the virus enters the cell through the TLR3 decorated endosomal compartment. TLR3 could thus be involved in recruitment of viral NC from the endosomal compartment, before aggregation in NBs. However, the absence of endosomal markers within the NBs remains unexplained. In addition, the presence of dsRNAs in NBs may suggest that NBs can also sequester dsRNAs and thus may play a role in the innate immune response to RABV infection.

Alternatively, given that TLR3 can trigger neuronal apoptosis [53], the sequestration of TLR3 could be seen as an attempt of RABV-infected neuronal cells to escape TLR3-induced apoptosis. Tanaka *et al*, 2004 showed an uncoupling of α-synuclein-/synphilin-1-positive aggregate formation and apoptosis [54]. They showed that aggregation of α-synuclein-/synphilin-1 seemed to promote cell survival rather than cell death. In our model, sequestration of TLR3 could be a way for the virus to prevent a TLR3-mediated pro-apoptotic response to infection, as neuronal integrity is required for RABV propagation though the CNS. Promotion of neuronal survival and virus replication are not exclusive and could be complementary strategies. Preliminary results indicate that RABV strains promoting either neuronal survival or death display different effects on NB size. Further experiments will be required to understand the role of NBs in the control of neuronal death.

In conclusion, these findings describe a novel role for TLR3 and describe how viruses — in our case, RABV — hijack normal functions of neuronal proteins and exploit cell compartmentalisation to favour the progression of their life cycle.

## Materials and Methods

### Cells and virus

Human Ntera-2clD/1 cells (ATCC CRL, 1973), human SK-N-SH cells (ATCC HTB11) and Hek293A (QBiogene) were grown in Dulbecco's Modified Eagle Medium (DMEM) with Glutamax-I, high glucose and sodium pyruvate 100 mM (Invitrogen, UK), supplemented with 10% FCS-N (foetal calf serum for neuronal cultures, Invitrogen, U.K.), 100 µg/ml streptomycin and 100 U/ml penicillin (Invitrogen). Human NT2-N cells [55] were differentiated from Ntera-2clD/1 cells by trans-retinoic acid and antimitotic treatment as previously described [56],[57]. BSR cells (a clone of BHK21 baby hamster kidney cells) [58] were cultivated in DMEM supplemented with 8% FCS. Human grade III U373MG astrocytoma (ATCC HTB 17) cells were propagated in Dulbecco's modified essential medium supplemented with 2 mM L-glutamine, 1 mM sodium pyruvate, 5% FCS, 2% sodium bicarbonate, 100 U/ml penicillin, and 100 µg/ml streptomycin at 37°C. The human microglial cell line CHME was cultivated as previously described [59]. The laboratory RABV strain CVS (ATCC vr959), a highly pathogenic strain causing fatal encephalomyelitis in mouse after intramuscular injection [60], was propagated as previously described [61]. Cells were infected at a multiplicity of infection (MOI) of 3 and cultivated for 12, 24 or 48 h at 37°C, in 5% CO2. HSV-1 strain KOS [62] was propagated in U373MG cells.

### Mice

TLR3^−/−^ mice were generated as described [2]. Six-week-old C57Bl6 from Janvier (St. Berthevin, France) or TLR3^−/−^ female mice were inoculated intramuscularly in both hind legs, with 1×10^7^ infectious particles of RABV. Disease progression was evaluated by scoring clinical signs and mortality. Mobility and mortality were scored as follows: 0 = normal mice, 1 = ruffled fur, 2 = one paralysed hind leg, 3 = two paralysed hind legs, 4 = total paralysis (defined as the total loss of mobility) and 5 = death. Daily clinical score was obtained by adding individual scores. Dead mice were reported in the clinical score of the day of the death and thereafter (cumulative scores). Disease progression was presented by a curve of cumulative clinical scores. Mortality was scored daily. Dead mice were counted on the day of death and thereafter (cumulative score). At day 11 after infection, groups of three mice were perfused with PBS. Brains were removed separately and stored at –80°C before being processed for RNA extraction. Animal housing and experimental protocols followed guidelines approved by the French Ministry of Agriculture and Ethical committee.

### Antibodies and reagents

Antibodies (Ab) to TLR3 (Q18, C20, H125) and anti-IgG goat biotinylated Ab were from Santa Cruz Biotechnology (Santa Cruz, CA, USA). Monoclonal antibodies (mAb) to TLR3 - IMG-315C (FITC-conjugated, immunostaining: 1/50), IMG-315A (Immunoblot: 1/500) and anti-TRIF (1/100) mouse polyclonal were from Imgenex (California, USA). Hoechst 33342, RIPA buffer, glutaraldehyde (G-5882), 20× SSC buffer, poly-D lysine and Ab to vimentin (Cy3 conjugate) (clone V9) and γ-tubulin (clone GTU-88) were from Sigma (Saint-Quentin Fallavier, France). Ab to Hsp70 (SPA-810, 1/200) was from Stressgen Bioreagents (Victoria, BC, Canada). Ab directed against calnexin (clone 37, 1/500) and EEA1 (1/500) were purchased at BD Transduction Laboratories. Fc Block™ and CellFIX™ were from BD Pharmingen (BD, Franklin Lakes, NJ, USA). KaryoMAX® Colcemid® Solution in PBS, Alexa Fluor® 488-conjugated anti-IgG rabbit and Alexa Fluor® 594-conjugated anti-IgG mouse were from Invitrogen (Cergy-Pontoise, France); ProLong® Gold Antifade reagent with or without DAPI and Alexa Fluor® 594-conjugated anti-IgG goat were purchased at Molecular Probes (Eugene, Oregon, USA). R-phycoerythrin-conjugated streptavidin was purchased from Dako (Trappes, France). Ab against CD63 is a kind gift from Eric Rubinstein (Inserm 602, Villejuif, France). Peroxidase-conjugated donkey anti-IgG mouse and AMCA-conjugated Streptavidin were from Jackson ImmunoResearch Laboratories (Suffolk, UK). For hybridisation experiments: RNA storage solution, proteinase K solution, deionised formamide, ultra pure BSA and yeast tRNA were from Ambion. Mouse mAb directed against the RABV P protein (721.2), RABV N protein (PVA-3) or the RABV G protein (PVE-12) have been previously described [63]–[65]. FITC-conjugated rabbit anti-RABV NC Ab and kaleidoscope prestained protein standard (161-0324) were obtained from Biorad (Marnes-La-Coquette, France). Streptavidin peroxidase, hybond-P PVDF (PolyVinylidine DiFluoride), biotinylated sheep anti-mouse IgG (RPN1001) and ECL^+^ kit were from GE Healthcare (UK). Micro BCA Protein Assay Reagent was from Pierce (Rockford, IL, USA). Ab against α-tubulin (clone DM1A, 1/1000) was from Oncogene Research Products, PhosphoSafe™ Extraction Buffer was from Novagen. Fluoromount-G was obtained from Southern Biotechnology (Birmingham, AL, USA). IgG2a mAb J2 (1/1000) directed against dsRNA, of length less than or equal to 40 bp, were obtained from English and Scientific Consulting Bt, Szirak, Hungary.

### RT–PCR, quantitative PCR

Total RNA was isolated from cells using an RNeasy kit (Qiagen, Germany). cDNA synthesis was performed with 2 µg RNA using oligo(dT) primers (100 ng). Superscript II reverse transcriptase (RT) and *Taq* DNA polymerase (QBiogene) were used for RT-PCR in a Px2 thermal cycler (Hybaid Corp., USA). 35 cycles of amplification were performed as follows: 4 min at 94°C, 1 min at 55°C, 1 min at 72°C, 10 min at 72°C, and final cooling. Ribosomal 18S RNA was used as a housekeeping gene. Primers used for amplification of the TLR3 and 18S genes were synthesised by Eurogentec (Belgium), with the following sequences (5′→3′): forward-18S: CTTAGAGGGACAAGTGGCG; reverse-18S: ACGCTGAGCCAGTCAGTGTA; forward-TLR3: GAGGCGGGTGTTTTTGAACTAGAA; reverse-TLR3: AAGTCAATTGTCAAAAATAGGCCT. The primer pair used for TLR3 exon IV deletion is (5′→3′): Forward-exon4-TLR3: CTCCAGGGTGTTTTCACGCAATTGG; Reverse-exon4-TLR3: TTCAGGTACCTCACATTGAAAAGCC. For quantitative PCR, specific primers for *TLR3* were from QIAGEN (QuantiTect Primer #QT00007714). For amplification of RABV genome the following primers were used (5′→3′): F = GGAATTCTCCGGAAGACTGGACCAGCTATGG;R = AGAATTCCCACTCAAGCCTAGTGAACGG. Real-time RT-PCR analysis was performed with an ABI Prism 77700 sequence detection system. Methods and relative quantification of gene expressions were carried out using the comparative method according to the manufacturer's instructions. Sequence of neuronal *TLR3* was determined and assigned the GenBank accession number DQ445682.

### Flow cytometry

RABV-infected and mock-infected NTera2clD/1 cells were washed once with phosphate-buffered saline (PBS) containing Ca^2+^Mg^2+^ (PBS Ca^2+^Mg^2+^), scraped (Cell Scraper, Corning) and pelleted in staining buffer (SB) (PBS, 1% inactivated FCS, 0.1% sodium azide, pH 7.5). Cells were fixed in 4% paraformaldehyde (PFA)/PBS for 30 min at 4°C, and resuspended in permeabilisation buffer (PB) (PBS, 1% FCS, 0.1% sodium azide, 0.1% saponin). Cells were then incubated with FITC-conjugated anti RABV NC Ab for detection of viral proteins and/or successively incubated with goat anti-TLR3 Ab (sc-Q18) followed by biotinylated anti-goat IgG Ab and finally with R-phycoerythrin-conjugated streptavidin. Cells were then washed with PB. For staining of non-fixed cells, cells were washed with SB instead of PB and fixed in CellFIX™ (BD Biosciences, USA). Cytofluorimetry was performed with a FACSCalibur™ (BD Biosciences). Results were analysed using the CellQuest™ Pro (BD Biosciences) software.

### Colcemid treatment

Cells were mock- or RABV-infected (at MOI 3) and treated or not at 24 h pi with colcemid (0.4 µg.ml^−1^) or vehicle alone (PBS). Cells were fixed at 48 h pi and immunostained for NC and tubulin proteins. Widefield observation was performed on Zeiss Apotome and Zeiss Axiovision 4.2 software for image acquisition. Nuclei were detected by DAPI staining and NB by anti-NC FITC Ab. Acapella software (PerkinElmer Acapella 2.0 (TM)) was used to delineate inclusions and measure of size of viral inclusions (area determined by number of pixels). The attribution of NB in each cell was computed by defining a region of interest around each nucleus. A set of negative controls has been used to fix the Acapella parameters (i.e. threshold or contrast adjustment). The different characteristics (area, position and number of NB for each cell) were saved and exported to Excel (Microsoft).

### Immunocytochemistry

RABV- and mock-infected cells were washed once with PBS Ca^2+^Mg^2+^, fixed with 4% PFA for 30 min at room temperature (rT), washed again, and treated with gelatin (1% in water) for 5 min at 4°C. The samples were then incubated for 20 min at rT in 0.3% Triton X-100-PBS and surface IgG receptors were blocked with a saturating buffer (ST) (2% bovine serum albumin and 5% FCS in PBS) for 30 min at rT followed by a 10 min incubation at 4°C with Fc-block (1/50). Viral NC were detected by incubation with a FITC-conjugated rabbit Ab for 2 h at rT. Primary and secondary Abs were diluted in ST. For immunostaining, cells were incubated with secondary Ab, biotin/ streptavidin, followed by incubation with DAB, resulting in a brown coloration. Nuclei were stained with Hoechst 33342 for immunofluorescence analysis or haematoxylin for transmitted light acquisition. Slides were washed with PBS Ca^2+^Mg^2+^, using water for the last wash. Coverslips were mounted in Fluoromount-G™ or ProLong Gold Antifade reagent (+/− DAPI if Hoechst previously used). Widefield observation was performed on a Leica DM 5000B UV microscope equipped with a DC 300FX camera (×40 and ×63 objectives). Leica FW 4000 software was used for image acquisition and processing. Confocal images were acquired using a Zeiss LSM 510 META (version 3.2) on Axiovert 200 M with a Plan Apochromat 63× and N.A. 1.4 objective. Confocal stacks were deconvoluted on Huygens® (Scientific Volume, Imaging, Netherlands) and 3D rendering was carried out using Imaris® (Bitplane AG, Switzerland) software. Confocal images were compiled using serial Z-stacks 0.3 µm apart, representing at least two experiments. Co-localisation of TLR3 and organelle markers was analysed using the colocalisation finder and threshold plugins of WCIF Image J. This allows quantification of the colocalisation ignoring pixels below the automatic thresholds.

### Immunoblotting

RABV- and mock-infected cells were washed once with PBS Ca^2+^Mg^2+^. Cells were lysed with PhosphoSafe™ Extraction or RIPA buffer. Soluble and insoluble cellular protein fractions were separated using previously described protocols [33],[42]. Lysate protein was quantified using the Micro BCA Protein Assay Reagent. Proteins (20 µg) were loaded into a 10% Tris/Glycine SDS polyacrylamide gel with Kaleidoscope pre-stained standards (161-0324, Biorad). Proteins were separated and transferred to a Hybond-P PVDF membrane for 2 h (150 mA), followed by saturation for 2 h at rT in saturating buffer (PBS-Tween 0.1%-Milk 5%). Membranes were incubated with primary Ab overnight at 4°C, washed in PBS-Tween 0.1%, then incubated with secondary Ab coupled to horseradish peroxidase (1 h at rT). Membranes were washed again and analysed by chemoluminescence using the ECL^+^ kit. Signals are acquired with a GBOX (Syngene) monitored by the Gene Snap (Syngene) software.

### Electron microscopy

Confluent RABV-infected or mock-infected SK-N-SH cells were cultured for 48 h at 37°C, in 5% CO2 on untreated 4 mm^2^ square glass coverslips. Cells were fixed for 30 min at 4°C in a PFA/glutaraldehyde solution. Culture medium was removed and immediately replaced by fixative solution with 2% freshly depolymerised PFA + 2% Glutaraldehyde in 0.1 M PBS. Subsequent treatments were performed at 4°C. Cells were rinsed three times in PBS and post-fixed for 30 min in 1% osmium tetroxide in milliQ water and rinsed. They were dehydrated in graded concentrations of ethanol followed by pure acetone before being infiltrated with Araldite. The resin was cured for 3 h at 37°C, then 36 h at 61°C. Ultrathin sections were obtained with a Reichert Ultracut. Sections were counterstained (or not) with uranyl acetate and lead citrate and observed on a Jeol 1010 electron microscope. Electron microscopy was also performed on cells fixed in 3% PFA, permeabilised with 0.1% Triton ×100, incubated with anti-RABV NC or anti-TLR3 (sc-H125) antibodies and revealed with DAB. Under these conditions, Abs can access intracellular targets, but cell structures are poorly conserved.

### *In situ* hybridization

Probe was designed to target the leader sequence genome of the RABV strain SAD B19 (5′ ACCAGATCAAAGAAAAAACAGACATTGTCAATTG 3′). RNA probe was synthesised by Dharmacon and a fluorescein molecule was added at the 3′ end. RNA probes were prepared according to Dharmacon's protocol and kept in the RNA storage solution. BSR cells were grown on poly-D lysine-coated slides and infected or not with RABV. Medium was removed at 36 h pi and cells were fixed for 30 min at 4°C with 4% PFA in PBS. All steps were carried out in RNAse-free conditions, except for the last washes. Cells were incubated for 5 min in 0.2 N HCl, rinsed in 2× SSC at rT and incubated for 10 min at 37°C with Proteinase K (20 µg/ml) in PBS. Slides were subjected to two series of washes in PBS and RNAse free-water, re-incubated for 10 min at 4°C with 4% PFA, dehydrated in graded ethanol solutions (70, 80, 90, 95 and 100%) for 1 min each and air dried. Cells were overlaid for 1 h at rT with pre-hybridisation solution (4 mM EDTA, 5× SSC, 50% deionised formamide, 100 µg/ml yeast tRNA, 0.5 mg/ml ultrapure BSA). Slides were then rinsed in 2× SSC for 1 min, drained and dried with a paper towel. Cells were incubated for 3 h at 55°C with fluorescein-conjugated RNA probe (20 ng/µl) diluted in hybridisation solution (4× SSC, 50% deionised formamide, 0,1% SDS, 8% dextran sulphate, 50 mM DTT) or with hybridisation solution only. Following hybridisation, cells were washed twice at RT in 2× SSC and 0.1× SSC for 10 min. Slides were then air-dried and mounted using ProLong® Antifade (Invitrogen). Samples were viewed using a Leica DM 5000B UV microscope and Leica TCS SP5 for confocal analysis.

### Silencing

*TLR3*-specific miRNA sequences cloned into pcDNA™6.2-GW/EmGFP-miR were purchased from Invitrogen (U.K.). High purity plasmid DNA was purified over JetStar columns (Genomed, Bad Oeyenhausen). For transient transfections, cells were grown to 80%–90% confluence and nucleofected using the Amaxa system as follows: HEK293A cells (Qbiogene) (10^6^) in 100 µl Nucleofector solution L (Amaxa) were mixed with 5 µg of the *TLR3*-specific miRNA or control (pcDNA™6.2-GW/EmGFP-miR-NEG) plasmid in a cuvette and subjected to Amaxa program Q-001 conditions. Nucleofected cells were grown in supplemented complete DMEM medium. 24 h post-nucleofection cells were incubated with blasticidin (5 µg/mL) and infected with RABV (MOI 3). 48 h pi cells were fixed and stained with the different Ab for further flow cytometry analysis. Total RNA extraction (RNeasy Kit, Qiagen, Germany) was also performed to determine the efficiency of silencing. Cells grown on slides were fixed with PFA 4% for further staining of TLR3 and viral NC proteins. Calculation of silencing efficiency was done as follows (cf TechNotes Volume 15(2), Applied Biosystems): the mean C_T_ were calculated for each condition and for both the reference gene (*18S*) and the gene of interest (*TLR3*). 2 values are calculated: ΔC_T_ ( = mean C_T_ (experimental ie *TLR3*)- mean C_T_ (endogenous ie *18S*)) and ΔΔC_T_ ( = ΔC_T_ (experimental)−ΔC_T_ (negative control)). The percent knockdown is obtained as follows: %KD = ([1–2^−ΔΔCT^]*100).

### Overexpression of a *TLR3* deletion construct

pUno-mcs and pZERO-hTLR3-(deltaTIR) plasmids (Invivogen) were amplified according to Invivogen protocol and purified using JetStar kit Plasmid purification MAXI kit (Genomed). DNA was quantified and plasmids used for nucleofection of Hek293 cells (Amaxa program Q-001) (5 µg plasmid for 1×10^6^ cells). 48 h post-nucleofection cells were infected with RABV and 24 h post-infection cells were harvested for total RNA extraction. Q-PCR was carried out as described above. Stable cell lines were established by addition of selective antibiotics (blasticidin or puromycin) to culture medium; cells were passaged over several weeks (≥5 weeks).

### Data analysis

Graphs (Figures 1C, 4D, 7B, 7C, 8A, 8B, 9, S4) and Student's T tests (Figures 4D left, 7B, 7C, 9, S4) were generated and performed using GraphPad Prism version 5.00 for Windows (GraphPad Software, San Diego California USA,). For mice experiments, collected data were plotted for comparison of Kaplan-Meier survival curves and statistical analysis was performed with MedCalc software.

## Supporting Information

Figure S1 TLR3 protein in NT2-N. Intracellular localisation of TLR3 in human post-mitotic neuron (NT2-N) cells. NI and RABV-infected NT2-N were immunostained with TLR3 and NC Abs. In the absence of infection (NI), TLR3 is localised in small vesicles throughout the cytoplasm. In RABV-infected cells, TLR3 and viral NC proteins are assembled in well-defined ovoid cytoplasmic structures (arrow). N = nuclei. Bars = 5 µm.
(1.50 MB TIF)

Figure S2 dsRNA can be detected within TLR3-positive viral NBs. Intracellular detection of dsRNA was assessed using J2 Ab (red) in non-infected (NI, upper panels) and RABV-infected SK-N-SH (lower panels). NBs are detected with anti viral NC Ab (24 h pi). dsRNA are found in both NI and RABV-infected cells and within the viral NBs, mainly in the corona.
(1.71 MB TIF)

Figure S3 NBs are TLR3/NC aggregates. Electron microscopy section of 48h-RABV infected SK-N-SH showing typical NBs containing viral NC (A) and TLR3 (B) proteins. NB = Negri Bodies. B.w = base of culture well. C.m = culture medium. Bars = 5 µm in A and 2 µm in B.
(1.70 MB TIF)

Figure S4 TIR is dispensable for NBs formation. TRIF - TLR3 adaptor - cannot be detected in NBs. (A) Overexpression of a TIR-deleted form of TLR3 (encoded by the pZERO-hTLR3-HA plasmid from Invivogen) was assessed by RT Q-PCR (left panel) in Hek293A cells. An average fold increase of 25 for TLR3 mRNA in cells transfected with pZERO plasmid was obtained compared to cells transfected with empty vector. Graph represents means and SD. (B) Overexpression of a deleted form of TLR3 does not modify the formation of viral NBs (arrows) as shown by immunostaining of cells expressing pZERO vector (right images) in comparison of cells with empty vector (left images). TLR3 was detected using Q18 Ab (red) and RABV using anti-NC Ab (FITC, green). Nuclei (blue) were stained with DAPI. Bar = 10 µm. (C) Immunostaining of RABV-infected SK-N-SH with an anti-TRIF (green), an anti- RABV P protein (red) Ab and Hoechst (Nuclei, blue) revealed that TRIF is not located within viral NBs. Bar = 5 µm.
(1.10 MB TIF)
