# Supplementary figures and images for: Toll-Like Receptor 3 (TLR3) Plays a Major Role in the Formation of Rabies Virus Negri Bodies
Source: PLoS Pathog. 2009 Feb 27;5(2):e1000315. doi: 10.1371/journal.ppat.1000315 (PMC2642728; doi:10.1371/journal.ppat.1000315)

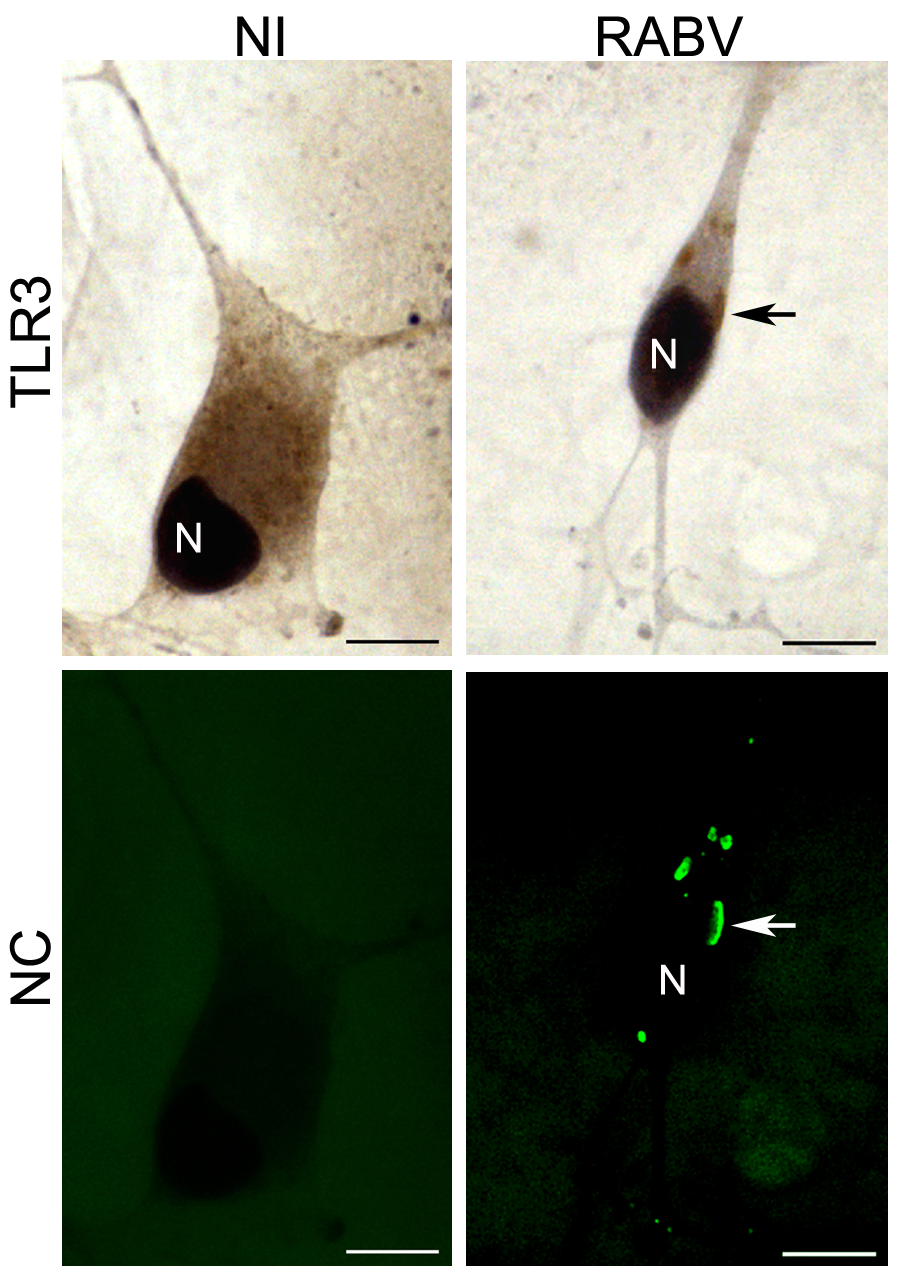

Supplement: Figure S1 — TLR3 protein in NT2-N. Intracellular localisation of TLR3 in human post-mitotic neuron (NT2-N) cells. NI and RABV-infected NT2-N were immunostained with TLR3 and NC Abs. In the absence of infection (NI), TLR3 is localised in small vesicles throughout the cytoplasm. In RABV-infected cells, TLR3 and viral NC proteins are assembled in well-defined ovoid cytoplasmic structures (arrow). N = nuclei. Bars = 5 µm. (1.50 MB TIF) [file ppat.1000315.s001.tif]

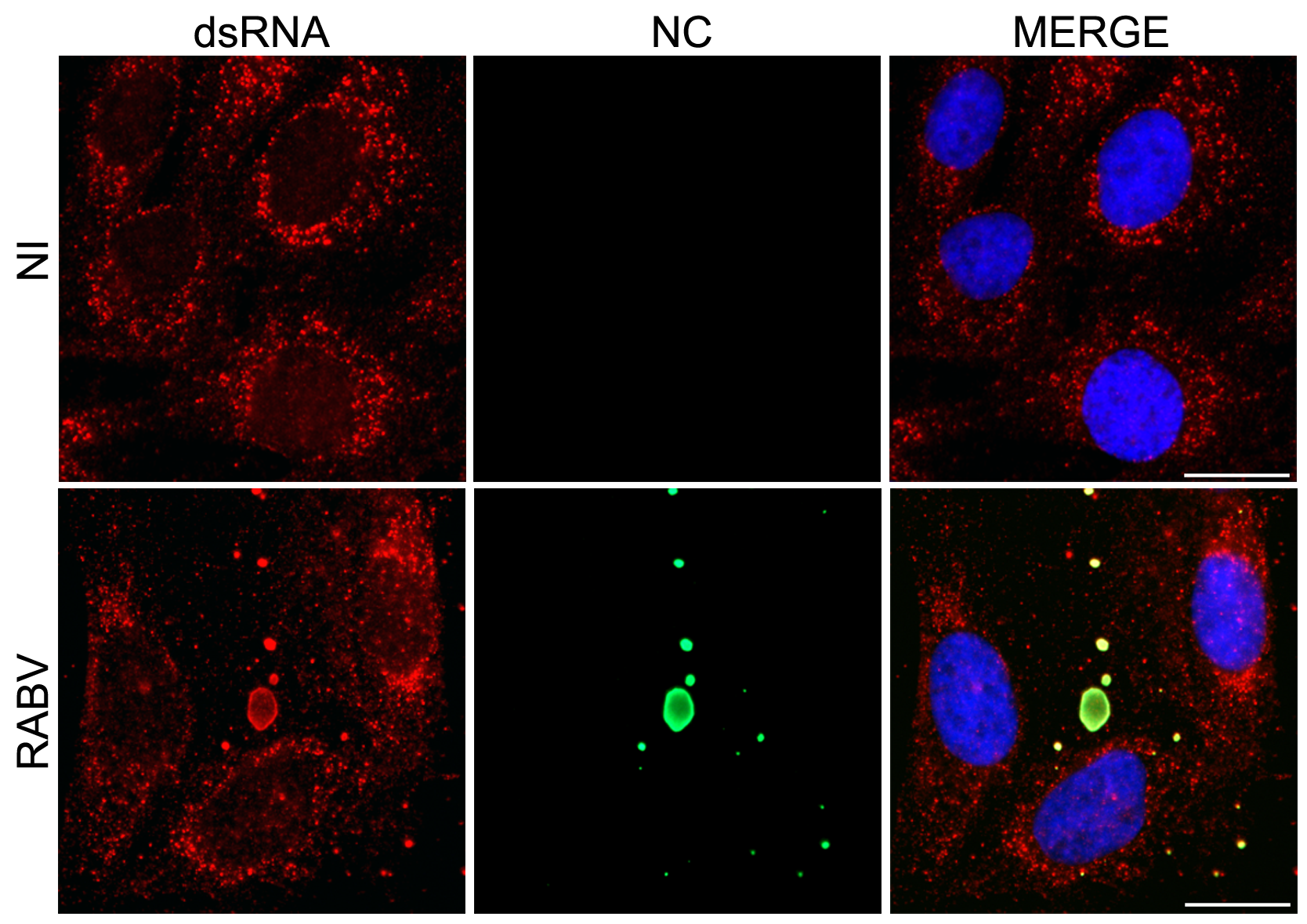

Supplement: Figure S2 — dsRNA can be detected within TLR3-positive viral NBs. Intracellular detection of dsRNA was assessed using J2 Ab (red) in non-infected (NI, upper panels) and RABV-infected SK-N-SH (lower panels). NBs are detected with anti viral NC Ab (24 h pi). dsRNA are found in both NI and RABV-infected cells and within the viral NBs, mainly in the corona. (1.71 MB TIF) [file ppat.1000315.s002.tif]

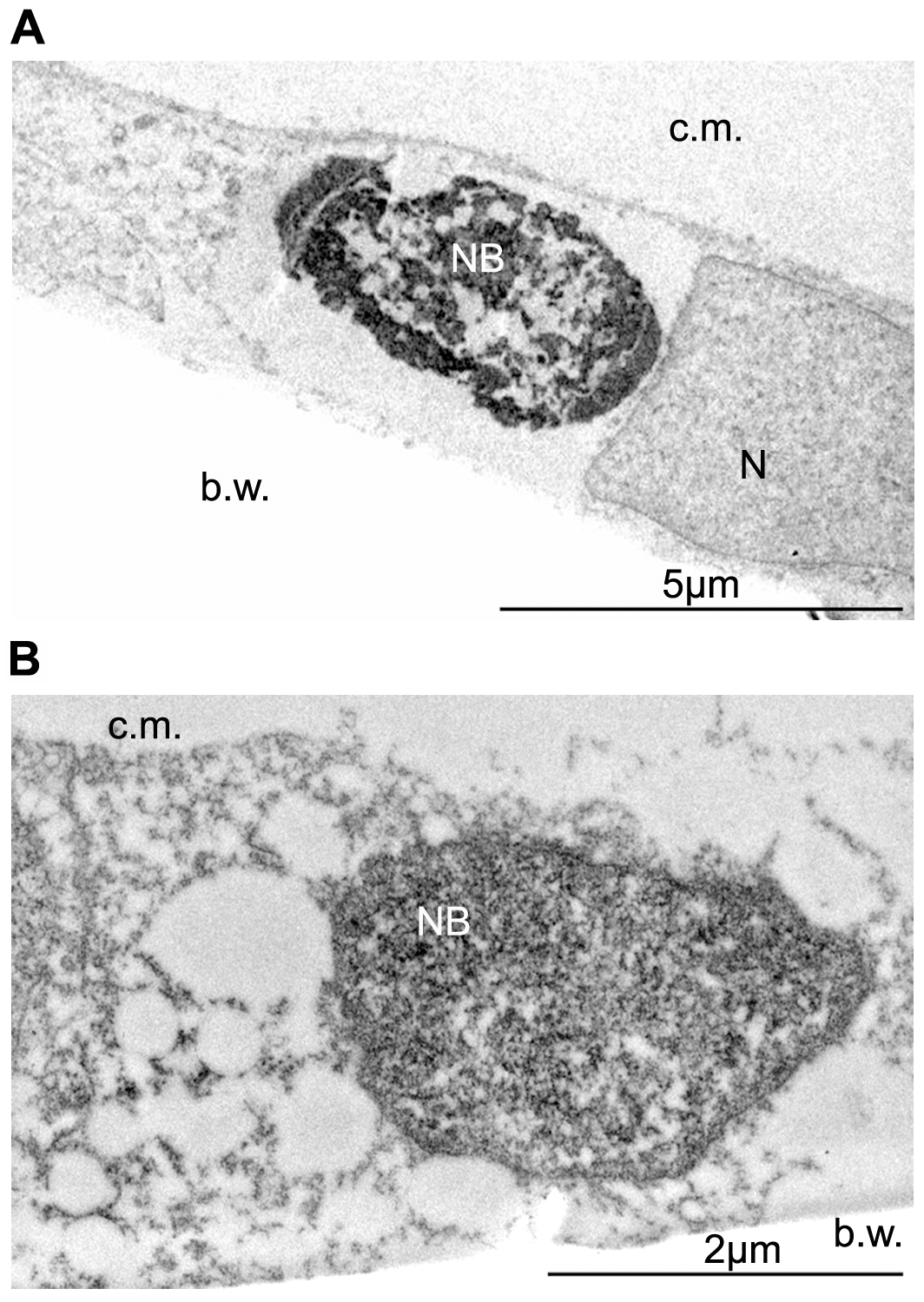

Supplement: Figure S3 — NBs are TLR3/NC aggregates. Electron microscopy section of 48h-RABV infected SK-N-SH showing typical NBs containing viral NC (A) and TLR3 (B) proteins. NB = Negri Bodies. B.w = base of culture well. C.m = culture medium. Bars = 5 µm in A and 2 µm in B. (1.70 MB TIF) [file ppat.1000315.s003.tif]

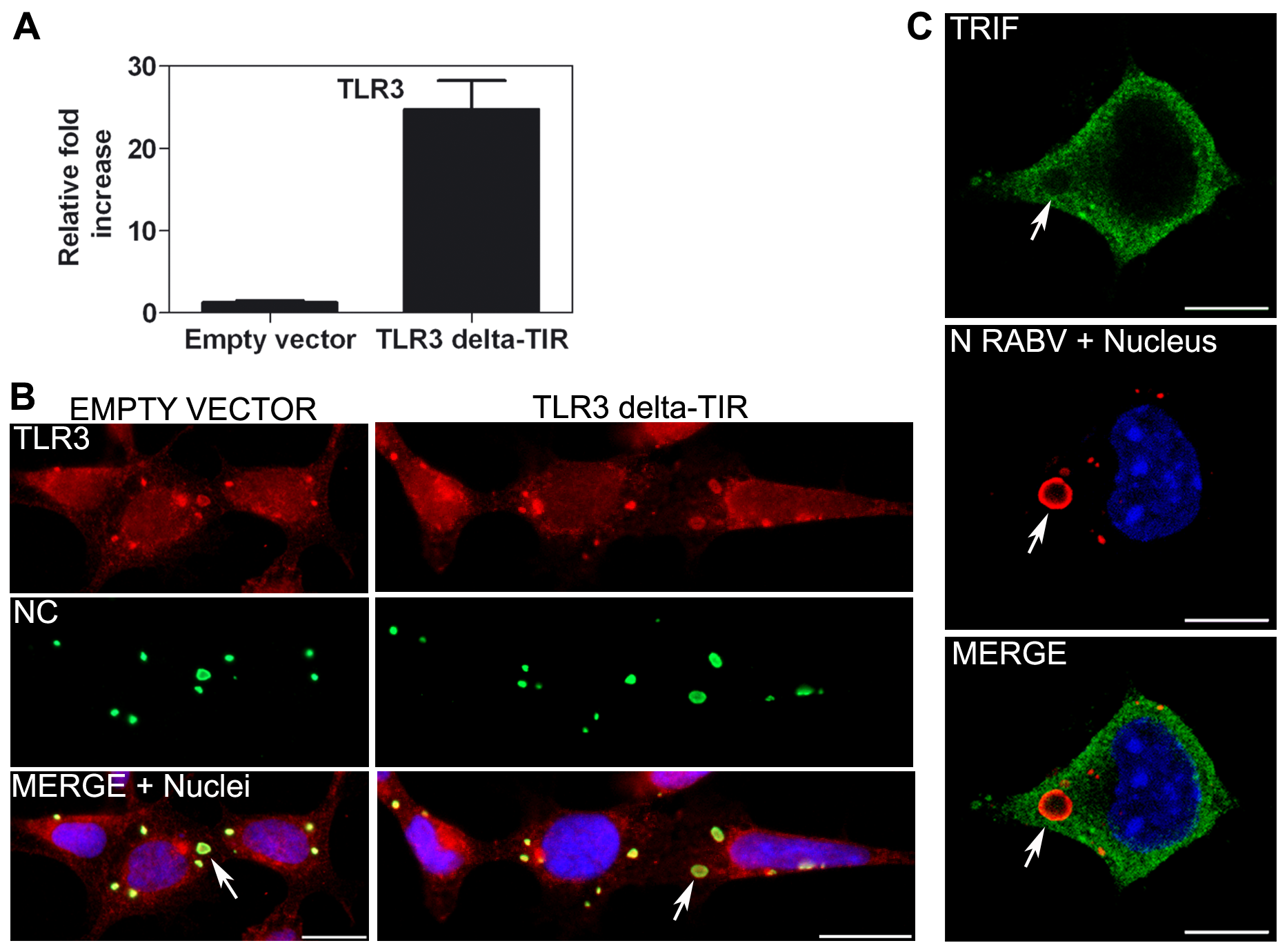

Supplement: Figure S4 — TIR is dispensable for NBs formation. TRIF - TLR3 adaptor - cannot be detected in NBs. (A) Overexpression of a TIR-deleted form of TLR3 (encoded by the pZERO-hTLR3-HA plasmid from Invivogen) was assessed by RT Q-PCR (left panel) in Hek293A cells. An average fold increase of 25 for TLR3 mRNA in cells transfected with pZERO plasmid was obtained compared to cells transfected with empty vector. Graph represents means and SD. (B) Overexpression of a deleted form of TLR3 does not modify the formation of viral NBs (arrows) as shown by immunostaining of cells expressing pZERO vector (right images) in comparison of cells with empty vector (left images). TLR3 was detected using Q18 Ab (red) and RABV using anti-NC Ab (FITC, green). Nuclei (blue) were stained with DAPI. Bar = 10 µm. (C) Immunostaining of RABV-infected SK-N-SH with an anti-TRIF (green), an anti- RABV P protein (red) Ab and Hoechst (Nuclei, blue) revealed that TRIF is not located within viral NBs. Bar = 5 µm. (1.10 MB TIF) [file ppat.1000315.s004.tif]
